# Practical guidelines for the use of gradient boosting for molecular property prediction

**DOI:** 10.1186/s13321-023-00743-7

**Published:** 2023-08-28

**Authors:** Davide Boldini, Francesca Grisoni, Daniel Kuhn, Lukas Friedrich, Stephan A. Sieber

**Affiliations:** 1 Department of Bioscience, Center for Functional Protein Assemblies (CPA), Technical University of Munich, Garching bei Munich, Germany; 2 Department of Biomedical Engineering, Institute for Complex Molecular Sciences, Eindhoven University of Technology, Eindhoven, The Netherlands; 3 Centre for Living Technologies, Alliance TU/E, WUR, UU, UMC Utrecht, Utrecht, The Netherlands; 4 Merck Healthcare KGaA, Darmstadt, Germany

**Keywords:** Gradient boosting, Virtual screening, QSAR

## Abstract

Decision tree ensembles are among the most robust, high-performing and computationally efficient machine learning approaches for quantitative structure–activity relationship (QSAR) modeling. Among them, gradient boosting has recently garnered particular attention, for its performance in data science competitions, virtual screening campaigns, and bioactivity prediction. However, different variants of gradient boosting exist, the most popular being XGBoost, LightGBM and CatBoost. Our study provides the first comprehensive comparison of these approaches for QSAR. To this end, we trained 157,590 gradient boosting models, which were evaluated on 16 datasets and 94 endpoints, comprising 1.4 million compounds in total. Our results show that XGBoost generally achieves the best predictive performance, while LightGBM requires the least training time, especially for larger datasets. In terms of feature importance, the models surprisingly rank molecular features differently, reflecting differences in regularization techniques and decision tree structures. Thus, expert knowledge must always be employed when evaluating data-driven explanations of bioactivity. Furthermore, our results show that the relevance of each hyperparameter varies greatly across datasets and that it is crucial to optimize as many hyperparameters as possible to maximize the predictive performance. In conclusion, our study provides the first set of guidelines for cheminformatics practitioners to effectively train, optimize and evaluate gradient boosting models for virtual screening and QSAR applications.

**Graphical abstract:**

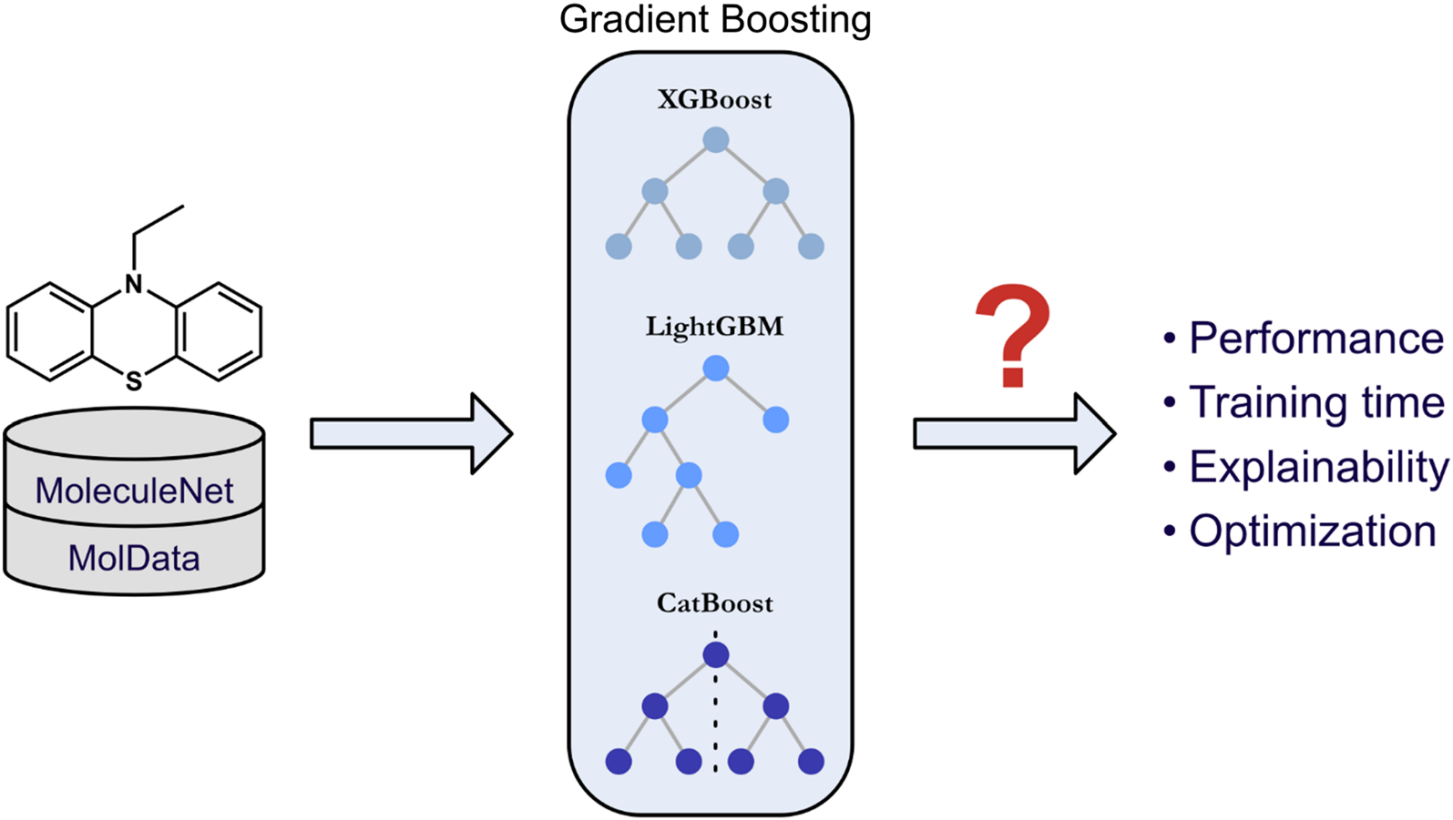

**Supplementary Information:**

The online version contains supplementary material available at 10.1186/s13321-023-00743-7.

## Introduction

Quantitative structure–activity relationship (QSAR) modelling occupies a vital role in cheminformatics research [1–5]. QSAR aims to link the molecular structure with experimentally measurable properties, and it is routinely used to predict molecular properties such as bioactivity [6–9], toxicity [10–13] and absorption, distribution, metabolism and excretion (ADME) [3, 14], thus covering a fundamental role in both hit discovery and hit-to-lead optimization.

QSAR aims to link the molecular structure (numerically encoded as the so-called molecular descriptors) [15–17] with experimentally measurable properties. For this application, decision tree ensembles are among the most used machine learning methods thanks to their excellent performance, ability to rank features in terms of importance and their ability to scale to large datasets [18, 19], alongside other popular frameworks like support vector machines (SVM) [20, 21].

Among decision tree ensembles, gradient boosting machines (GBM) have seen a strong surge in popularity in the last years, driven by excellent results in data science competitions and state-of-the-art performance in modelling tabular data [22, 23]. GBM iteratively aggregates predictive models so that each one compensates the errors from the previous step, thus yielding a high-performance ensemble.

In cheminformatics, GBM has already found widespread use in several QSAR tasks such as toxicity prediction [12], drug sensitivity analysis [24], anti-cancer activity modelling [25] and drug-target interaction identification [26], as well as showing competitive performance with deep learning approaches in recent large-scale benchmarking studies [16, 27–30].

However, several implementations of the GBM algorithm exist, each with unique modifications to the original formulation and employing different decision tree structures [23], such as XGBoost [31], LightGBM [32] and CatBoost. [33] While the importance of these differences has been recognised in other fields [23], these algorithms are used interchangeably in chemoinformatics, and to our knowledge their respective advantages are not well documented. Thus, there is an urgent need for a rigorous benchmarking of these different implementations for QSAR applications. This is further warranted by the uniqueness of cheminformatics datasets compared to other typical tabular datasets like finance and real estate price prediction [22, 23]. For example, datasets in this field tend to have a much higher number of features, they are often extremely imbalanced [34] and might contain false positives or false negatives [35].

The aim of this paper is to provide the first set of practical guidelines for the use of gradient boosting in QSAR applications, such as toxicology and drug discovery, by answering the following questions:

1. Which gradient boosting implementation performs the best for QSAR?
2. Which package scales the best to large datasets, such as high throughput screens (HTS)?
3. Do they produce similar feature importance rankings, or do they highlight different molecular features?
4. Is it possible to identify the most important hyperparameters to optimize for these algorithms to accelerate further the development and deployment of these methods for QSAR?

To answer these questions, we carried out a large-scale benchmark of these three implementations on 16 classification and regression datasets with 94 different endpoints commonly considered for virtual screening, covering a wide range of dataset size and class-imbalance ratios. To ensure the robustness of our results, we extensively optimized each algorithm according to the guidelines set up by the respective authors of the packages and recent studies, constructing 157,590 individual QSAR models.

## Methods

GBM is an ensemble algorithm, which aims to aggregate several decision trees into a single more performant predictor. Decision trees are a machine-learning algorithm that learns a flowchart-like structure of hierarchical binary decisions [36]. The terminal nodes of the graph are generally named leaves, which are used to assign sample predictions [36]. To explain how GBM constructs the decision tree ensemble, we first present the original implementation of the algorithm [37] followed by a systematic analysis of the changes introduced by XGBoost, LightGBM and CatBoost.

### Gradient boosting

Given an input matrix $X$ and a vector $Y$ of molecular properties (e.g., biological activity), the gradient boosting algorithm approximates the underlying function $F\left(x\right)$, which maps the relationship between the molecular descriptor ${x}_{i}$ and the biological activity ${y}_{i}$, with a function $\hat{F}\left(x\right)$, constructed in an additive manner:

1 $$\begin{array}{*{20}c} {\hat{F}\left( x \right) = \,\sum\limits_{{m = 1}}^{M} {\sigma *\widehat{{F_{m} }}\left( x \right)} } \\ \end{array}$$

where $\sigma$ is the learning rate, a constant regularization parameter limiting the influence of a given predictor within the ensemble, and $\widehat{{F}_{m}}\left(x\right)$ is the $m$th tree. Given a loss function $L\left({y}_{i},{p}_{i}\right)$, such as the binary cross-entropy, that measures the quality of predictions ${p}_{i}$ with respect to real readouts ${y}_{i}$, after the first iteration each new tree $\widehat{{F}_{m}}$ is learned by minimizing the following objective:

2 $$\begin{array}{c}\widehat{{F}_{m}}=argminE\left(\frac{-\partial L\left(Y,{P}_{m-1}\right)}{\partial {P}_{m-1}}-{P}_{m}\right)\end{array}$$

where the derivative of the loss with respect to the ensemble output represents the prediction residuals of $\hat{F}\left(x\right)$ at the previous iteration, and ${P}_{m}$ are the predictions at the current iteration. As such, each new decision tree is constructed so that it compensates the prediction errors of the model during the previous iteration, essentially conducting gradient descent in function space instead of parameter space.

The original formulation of GBM is the one employed by the popular machine learning package Scikit-learn [38]. Unfortunately, this implementation lacks many of the regularization and optimization methods implemented by XGBoost, CatBoost and LightGBM and cannot be parallelized across multiple CPU cores. For this reason, we did not include the Scikit-learn version of GBM in the benchmarking study.

### XGBoost

XGBoost introduces a regularized learning objective [31]. At a given iteration $m$, instead of being computed according to the loss function $L\left({y}_{i},{p}_{i}\right)$, the residuals are calculated with the following formula:

3 $$\begin{array}{*{20}c} {L_{\emptyset } \left( {y,p} \right) = \sum\limits _{{i = 1}}^{I} L\left( {y_{i} ,p_{i} } \right) + \gamma T_{m} + \frac{1}{2}\lambda \left\| {w_{m} } \right\|^{2} } \\ \end{array}$$

where $\gamma$ and $\lambda$ are regularization hyperparameters, ${T}_{m}$ is the number of leaves in the $m$th tree and ${\Vert {w}_{m}\Vert }^{2}$ is the L2 norm of its leaf weights. Thanks to this modification, XGBoost learns simpler trees with smoother weights, which leads to better generalization [31]. Additionally, XGBoost employs Newton descent instead of gradient descent to optimize its trees, which leads to faster convergence [39]. Finally, XGBoost also introduced a new feature split finding algorithm to speed up training [31].

### LightGBM

**Fig. 1:**
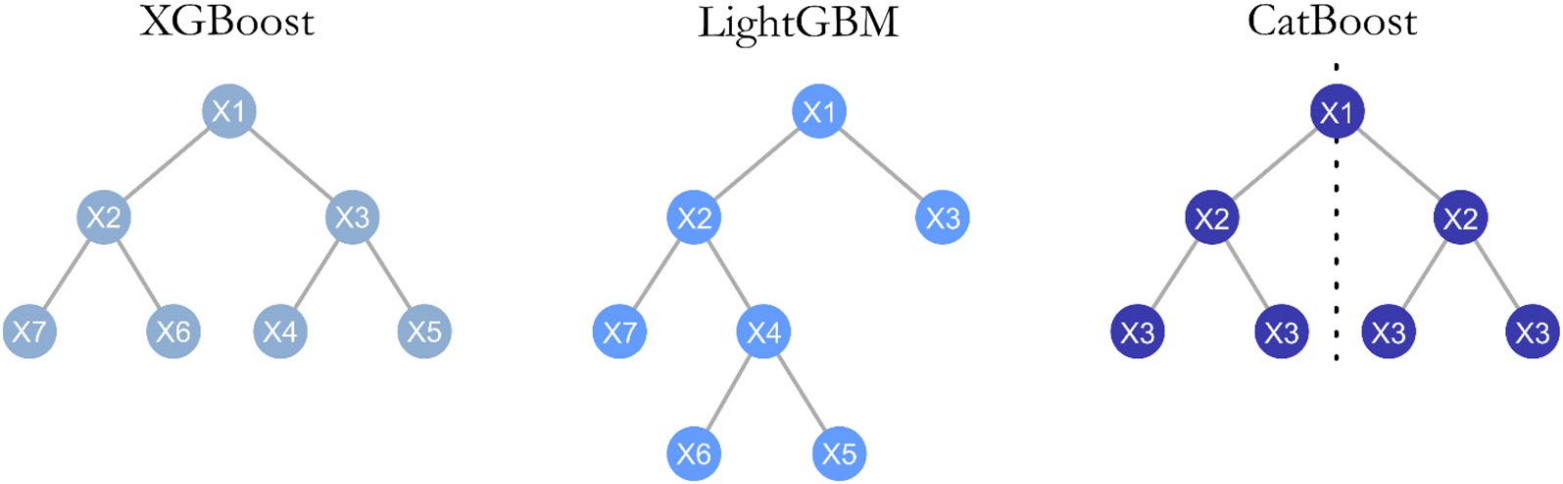
Different tree structures and split indexes (shown inside each node) generated by XGBoost, LightGBM and CatBoost. XGBoost adopts a “breadth-first” search, maintaining constant tree depth across branches. LightGBM uses a “depth-first” criterion, yielding asymmetric trees. CatBoost relies on oblivious trees, where at a given depth the same split is used across all branches, as indicated by the constant split indexes

This implementation also adopts many solutions proposed by XGBoost to improve the performance such as the regularized learning objective and Newton descent. However, LightGBM introduces three new strategies to make training more efficient: a histogram-based split finding method, Exclusive Feature Bundling (EFB) and Gradient-based One-Side Sampling (GOSS) [32]. EFB employs heuristics to find groups of mutually exclusive features and merges them together, thus reducing the dimensionality of the dataset, while GOSS relies on gradients to sample at each iteration the most important dataset instances without changing the training set distribution. Each of these algorithms simplifies different aspects of the original minimization objective, thus speeding up training time with negligible loss in accuracy. Furthermore, LightGBM employs a different tree growth strategy compared to XGBoost. In most cases, trees are generated in a “breadth-first” fashion, where every time a new split is found, all other splits at the same level are first considered before increasing further the depth of the tree. This yields tree structures that have the same depth across all branches. In contrast, LightGBM grows trees in a “depth-first” fashion (Fig. 1), where the algorithm splits nodes exclusively according to the largest performance gain [40]. This procedure leads to asymmetric trees, where certain branches might be very deep while others might be shallow. This approach tends to converge faster, but might be susceptible to overfitting on small datasets [32].

### Catboost

There are three main features that distinguish CatBoost from LightGBM and XGBoost. First, it provides a novel Target Statistics (TS) algorithm to handle categorical variables, which leads to more robust performance on unseen data by addressing the issue of target leakage during training [33]. However, categorical inputs are very rarely found in molecular descriptors [41], therefore this aspect is not of big relevance for cheminformatics applications. Second, it introduced ordered boosting, a variation of gradient boosting where each model is trained on a different partition of the training dataset, tackling the issue of prediction shift that arises by fitting trees on gradients obtained from samples already used during training. In principle, this approach reduces the risk of overfitting, especially on small datasets [33]. Third, CatBoost employs “oblivious decision trees”, where the same variable and threshold are used to generate splits at a given depth level (Fig. 1) [33, 42]. This enforced symmetry acts as regularization, constraining the expressiveness of tree models, and can be leveraged to provide uncertainty estimates on predictions, similarly to Gaussian Processes models [43]. Finally, the authors of this library have researched extensively the theoretical properties of gradient boosting and proposed several new features like Langevin gradient descent [44] and sample importance analysis [45], which are only available in the CatBoost package [42].

## Experiments

### Datasets

To provide a robust evaluation framework for our benchmark analysis, we evaluated XGBoost, LightGBM and CatBoost on 16 classification and regression datasets from three well-established repositories: MoleculeNet, [27] MolData [1] and the ChEMBL benchmarking study from Cortés-Ciriano et al [46] (Table 1). From the first, we included Tox21, MUV, HIV, ClinTox, BBBP, BACE and SIDER. From the second, we chose the Phosphatase, NTPase, Oxidoreductase and Fungal datasets. From the third, we selected HERG, Acetylcholinesterase, COX-2, erbB1 and JAK-2. We retrieved the MoleculeNet datasets from a recent benchmarking study [16], while we referred to the original publications for the MolData repository and the ChEMBL datasets [1, 46]. This selection entails approximately 1.4 million unique compounds and 94 endpoints on a wide variety of protein families and biological responses, ensuring that our findings are broadly applicable for cheminformatics applications. Our selection covers an extensive range of compounds per endpoint (from 2000 to 330,000) and imbalance ratios between compounds classified as either ‘positive’ or ‘negative' (from 1:2 to 1:500), reflecting the diversity of datasets typically encountered in cheminformatics (Table 1).

**Table 1:** Datasets employed in this study. For datasets with multiple endpoints, we reported the ranges between minimum and maximum values regarding the compounds per endpoint and imbalance ratios

Name	Type	Source	Endpoints	Compounds per endpoint	Class imbalance ratio
Tox21	Classification	MoleculeNet	12	5810–7265	1:5–1:33
HIV	Classification	MoleculeNet	1	40,748	1:27
MUV	Classification	MoleculeNet	17	14,415–14,903	1:486–1:613
BACE	Classification	MoleculeNet	1	1513	1:1
BBBP	Classification	MoleculeNet	1	2039	1:3
SIDER	Classification	MoleculeNet	27	1427	1:12–1:63
ClinTox	Classification	MoleculeNet	2	1478	1:12–1:14
Phosphatase	Classification	MolData	5	260,322–298,215	1:121–1:576
NTPase	Classification	MolData	6	251,895–301,932	1:3–1:16,265
Oxidoreductase	Classification	MolData	10	79,853–325,083	1:9–1:9847
Fungal	Classification	MolData	7	152,880–302,256	1:135–1:640
HERG	Regression	Cortés-Ciriano et al.	1	5207	N.A
Acetylcholinesterase	Regression	Cortés-Ciriano et al.	1	3159	N.A
COX-2	Regression	Cortés-Ciriano et al.	1	2855	N.A
erbB1	Regression	Cortés-Ciriano et al.	1	4868	N.A
JAK-2	Regression	Cortés-Ciriano et al.		2655	N.A

### Performance metrics

For each classification dataset, we evaluated the Receiver Operating Characteristic Area Under Curve (ROC-AUC) and Precision-Recall Area Under Curve (PR-AUC). Our selection is consistent with the figures of merit used in the literature when evaluating these datasets and ensures that the results are not skewed by high imbalance ratios [1, 16, 27, 47, 48]. For the regression datasets, we evaluated the Root Mean Squared Error (RMSE). To assess whether differences in performance are statistically significant, we used the two-tailed Mann–Whitney test with Bonferroni correction [49].

### Molecular descriptors

We featurized all compounds using the Extended-Connectivity Fingerprints (ECFP) with radius of 2 and bit size of 1024 [50]. To ensure that bit collision is not a factor in any of our findings, we have investigated the change in vector sparsity when using larger bit sizes. Given that the number of unique fragments remains approximately constant for all datasets when increasing the bit size (Additional file 1: Table S1), we can exclude that bit collision plays a role for the benchmarks in this study.

### Performance analysis

We used three different optimization and evaluation protocols, depending on whether the dataset is from MoleculeNet, MolData or ChEMBL. The reason for this is to keep our analysis consistent with prior studies from the scientific literature, and because the datasets from MolData are several orders of magnitude larger than the ones in the MoleculeNet repository or from Cortés-Ciriano et al [46].

For MoleculeNet datasets, we replicated a previously proposed procedure [16], whereby for each endpoint, each classifier is optimized with Hyperopt [51] for 100 iterations using an extensive hyperparameter grid, determined according to existing guidelines and benchmarks [22, 39, 40, 42]. The full hyperparameter grid is available in the Supporting Information. Each optimization iteration measured the average PR-AUC with a given hyperparameter setting across three random train-test splits with an 80:20 ratio. Then, the model was run with the optimal hyperparameters on 50 independent evaluations with random splits, using the same ratio between training and test set. After each run, the ROC-AUC and PR-AUC were measured on the test set as well as the training time. Finally, for a given dataset, the performance metrics and training times were averaged across replicates and across endpoints.

For the MolData benchmarks, we used the scaffold splits provided by Arshadi and coworkers during optimization and evaluation [1]. As such, for each endpoint, each classifier was optimized for 100 iterations using Hyperopt [51] with the same grid as above. Each iteration measured the PR-AUC obtained by the classifier with a given hyperparameter setting on the validation set. Then, the model was run with optimal hyperparameters on five independent evaluations with different random seeds, measuring the ROC-AUC and PR-AUC on the test set as well as the training time. As above, the results were reported as averages across replicates and endpoint for a given dataset.

For the regression datasets from Cortés-Ciriano et al. [46], we adopted the procedure employed in the original publication. In short, each dataset was split into training, validation and test sets with a 70:15:15 ratio using random splits. We then performed hyperparameter tuning via Hyperopt, optimizing RMSE on the validation split for 100 iterations, using the same grid as above. Finally, we repeated training on the training split and evaluation of RMSE on the test set for 50 iterations. As such, the final RMSE values were indicated as averages across replicates for each dataset.

### Feature ranking analysis

One of the advantages of GBM is that it can provide information on the feature importance, which can be used as a tool to provide indication of what drives the model predictions, and, in certain cases, to achieve model explainability [52]. We used Shapley values [19, 53] to assess which molecular features are the most important according to each GBM predictor. Shapley values quantify the importance of each feature (‘feature attribution’ [37]) by evaluating the change in a model’s predictions across all possible permutations [19, 52]. To obtain feature rankings for each dataset, we collected the Shapley values from each model with optimal hyperparameters during the evaluation procedure. Then, we averaged them across independent runs and dataset endpoint, obtaining one ranked list of variables per dataset for each model. To compare the variable rankings between pairs of GBM implementations, we employed the following formula:

4 $$\begin{array}{c}Overlap{\%}=\left(1-\frac{{{V}_{s}}_{k}}{k}\right)*100,k=20\end{array}$$

where $k$ is the cut-off for the number of most important variables to consider (set to *k* = 20 in the present study) and ${{V}_{s}}_{k}$ is the number of unique variables when considering both importance rankings. Intuitively, this metric measures the agreement of the two rankings, irrespective of the specific ordering, among the top 20 most important variables. For example, a score of 50 indicates that two GBM models have 10 molecular features in common when looking at their respective top 20 most important variables, regardless of whether these 10 features received the same rank in both lists. This score therefore shows whether the use of different gradient boosting algorithms would highlight the same features as most important, without being influenced by the ranking of less informative variables. However, it should be kept in mind that for many molecular representations such as hashed fingerprints, translating feature importance rankings into chemical insights is not a trivial task [54].

Finally, to evaluate the influence of converging to different hyperparameter configurations, regardless of algorithmic differences in the gradient boosting implementation, we also evaluated the feature ranking overlap between two independent LightGBM optimization runs. The analysis was limited to LightGBM due to computational costs and that considering one GBM is sufficient to evaluate the variability in feature ranking overlap induced by the stochasticity in the hyperparameter optimization process.

### Hyperparameter analysis

To evaluate the influence of each hyperparameter on the optimization process, we employed the Functional ANOVA (fANOVA) [55]. To acquire a sufficient collection of hyperparameter combinations, we optimized LightGBM with Hyperopt for 500 iterations on each endpoint, using the same hyperparameter grid and evaluation criteria as above. Because of the high computational cost for this analysis, we limited our study only to one GBM implementation and exclusively to classification datasets. Then, after pruning the worst 150 iterations, we processed the resulting parameter-performance pairs using fANOVA, yielding individual hyperparameter importance scores and their first-order interactions. By limiting the analysis to well-performing configurations, we ensured that the importance estimates for the parameters reflect their importance on reaching the optimum, and not on causing large oscillations in performance [55]. We excluded the SIDER and Fungal datasets from this analysis, since they were reserved as test sets to evaluate whether selecting hyperparameters according to their fANOVA importance score generalizes to unseen datasets. Furthermore, to assess the influence of molecular descriptors on the optimal hyperparameters, we also repeated this procedure using the MACCS keys [56] and an assortment of 207 physical–chemical descriptors from RDKit as featurization options. The complete list of descriptors is available in the Supporting Information (Additional file 1: Table S2).

### Software and implementation

Molecular descriptors were computed using RDKit (Version 2022.09.4) for python. [50] For training the models, XGBoost (Version 1.7.1) [39], LightGBM (Version 3.3.5) [40] and CatBoost (Version 1.1.1) [42] were employed. Scikit-learn (Version 1.2.1) [38] was used to split the MoleculeNet datasets and compute ROC-AUC and PR-AUC values. Each model was tuned via Bayesian hyperparameter optimization using the Hyperopt package (Version 0.2.7) [51]. Finally, SHAP (Version 0.41.0) [19] was utilized to compute Shapley values and the fANOVA package (Version 2.0.5) [55] was employed for the hyperparameter importance analysis. All calculations were performed on an AMD Ryzen Threadripper 3970X CPU with 32 cores and 64 threads. Training of the gradient boosting models was parallelized across all cores available. The code to reproduce the results is available at https://github.com/dahvida/GBM_Benchmarking.

## Results and discussion

### Predictive performance

**Fig. 2:**
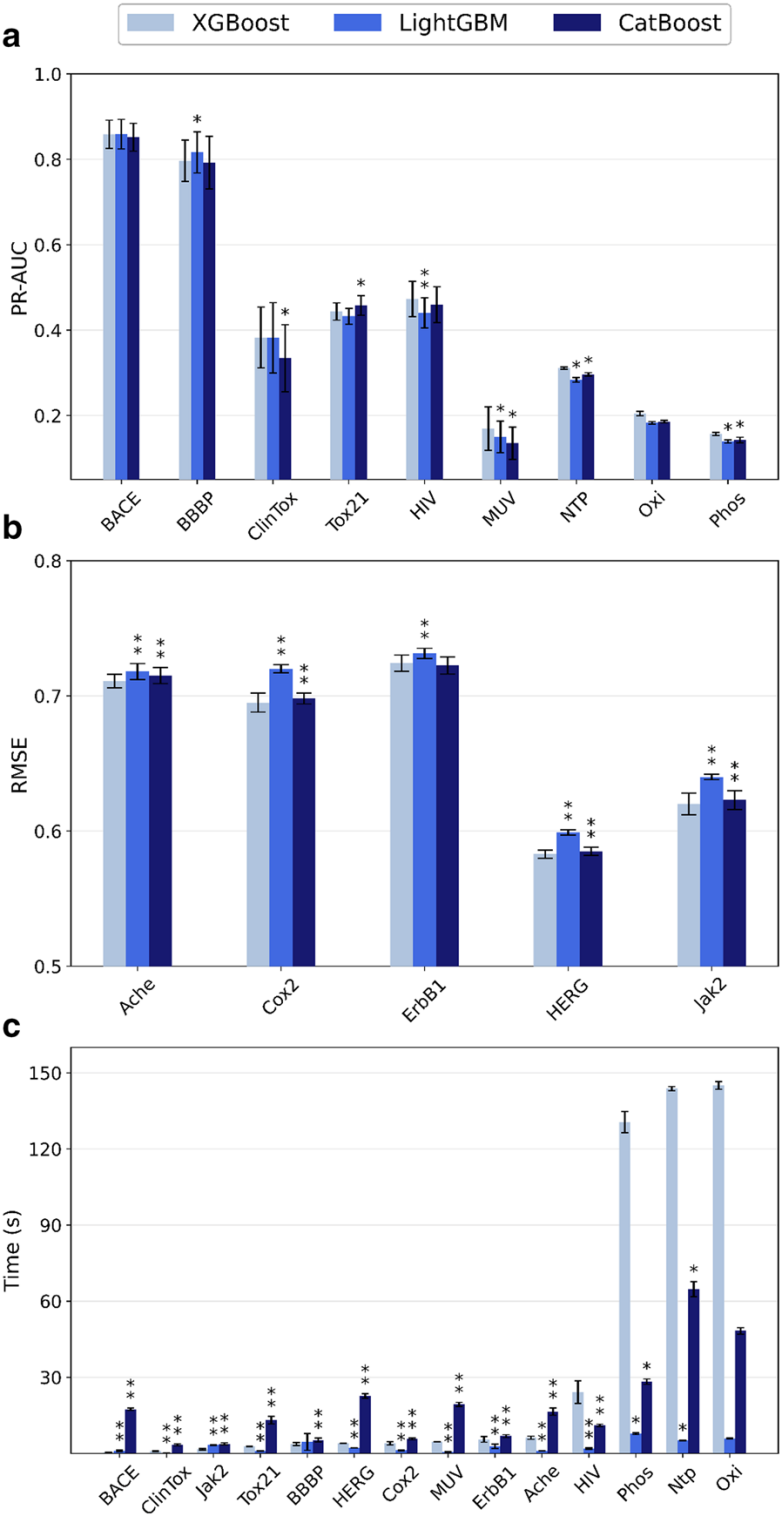
Performance comparison of all gradient boosting implementations in terms of **a** PR-AUC, **b** RMSE and **c** training time. All calculations were performed on an AMD Ryzen Threadripper 3970X CPU. Statistical tests are carried out with respect to XGBoost. Error bars represent the standard deviation (*N* = 50 for MoleculeNet datasets, *N* = 5 for MolData datasets), while the asterisks denote whether the difference is significant (*: α < 0.05, **: α < 0.01, with Bonferroni correction)

Overall, XGBoost achieves the best performance on most of the datasets (Fig. 2a, b and Additional file 1: Figure S1), with statistically significant differences in most cases. Interestingly, there seems to be a correlation between the improvement provided by XGBoost over the alternatives and dataset size. For smaller classification datasets (e.g., BACE, BBBP and ClinTox), CatBoost performs worse, with LightGBM being able to match or outperform XGBoost. This aspect is seemingly in contradiction with the concerns of overfitting due to its depth-first tree structure reported elsewhere. [40] For medium-sized datasets (e.g., Tox21, MUV and HIV, ranging from approximately 7000 compounds to 40,000), CatBoost tends to perform better than LightGBM, and it outperforms XGBoost on the Tox21 dataset. Finally, for large datasets (NTPase, Phosphatase and Oxidoreductase datasets, having more than 300,000 molecules per endpoint), XGBoost outperforms both LightGBM and CatBoost. When considering all datasets, XGBoost provides roughly a 5% improvement on average over LightGBM and CatBoost in terms of ROC-AUC and PR-AUC.

Regarding the regression datasets, LightGBM tends to achieve worse RMSE scores, while XGBoost ranks as the best performing algorithm on most benchmarks (Fig. 2b). CatBoost is generally able to match the performance of XGBoost, although the differences are statistically significant.

When considering the training times across all datasets (Fig. 2c), a similar dependence on the dataset size can be observed. LightGBM is the fastest algorithm on all benchmarks, due to the algorithm’s focus on reducing computational load. CatBoost is the slowest algorithm for small and medium sized datasets, while XGBoost requires significantly more time to train for larger datasets than both alternatives. While the absolute difference of training times for a single model is not particularly great (i.e., 5 versus 140 s on a CPU with 32 cores), it can significantly impact hyperparameter optimization procedures, where the model needs to be retrained many times. Furthermore, this difference will also grow significantly if less cores are available for training.

In summary, XGBoost provides the best predictive performance for cheminformatics out of all gradient boosting implementations, at the cost of training speed for larger datasets. LightGBM and CatBoost have comparable performance, but the former provides substantial benefits in terms of training time over the other algorithms.

### Feature ranking comparison

**Fig. 3:**
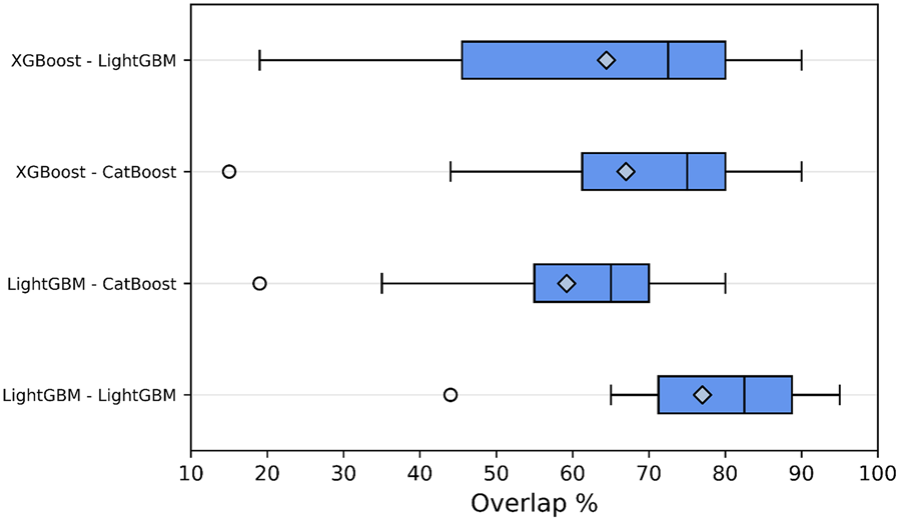
Box-plot distribution of overlap scores across all datasets for each gradient boosting implementation pair. The length of the box denotes the interquartile range, the diamond indicates the mean and the horizontal line defines the median. The comparison between two independent optimization runs using the same algorithm was limited to LightGBM due to its computational cost

We observed a remarkable variability between the importance rankings across different implementations, especially when comparing them to the overlap scores of two independent optimization and training runs for the same GBM algorithm (Fig. 3). For MUV, for example, there is approximately only a 20% overlap for any implementation pair, while for other datasets the agreement reaches up to 90%. The reason for the variability across implementations could be due to the use of different tree structures, as well as converging to different hyperparameter optima. For example, tuning the minimum split gain can lead to the selection of different splits, which in turn would yield different variable importance scores. This would explain the results obtained when comparing two runs of the same GBM algorithm across all datasets, since even in that scenario the variable overlap scores are distributed between 70 and 90% (Fig. 3). Another possible explanation for this pattern is that the algorithms highlight similar molecular fragments, but those fragments are mapped to different bits in the ECFP representation, thus producing semantically similar rankings despite not focusing on the same variables. To investigate this hypothesis, we calculated the top 20 ranked fragments for all GBM algorithms for the BACE datasets and manually inspected them (Additional file 1: Figure S2). When comparing the most important fragments between pairs of GBM predictor, each model had approximately ten unique substructures, which did not have any analogues in the other rankings. As such, it seems that each implementation indeed generates semantically distinct explanations for a given dataset, highlighting potential differences in the learned structure–activity relationships.

The main takeaway from this analysis is that using gradient boosting to evaluate which molecular features or fragments are the most influential is a non-trivial task, given the low agreement between different implementations of the same algorithm. Expert knowledge must always be employed to evaluate each fingerprint bit or molecular descriptor and to assess whether the explanations provided by the model are reasonable. Finally, averaging the Shapley scores on different hyperparameter optima or across different gradient boosting implementations might yield better estimates of feature importance.

### Hyperparameter importance

**Fig. 4:**
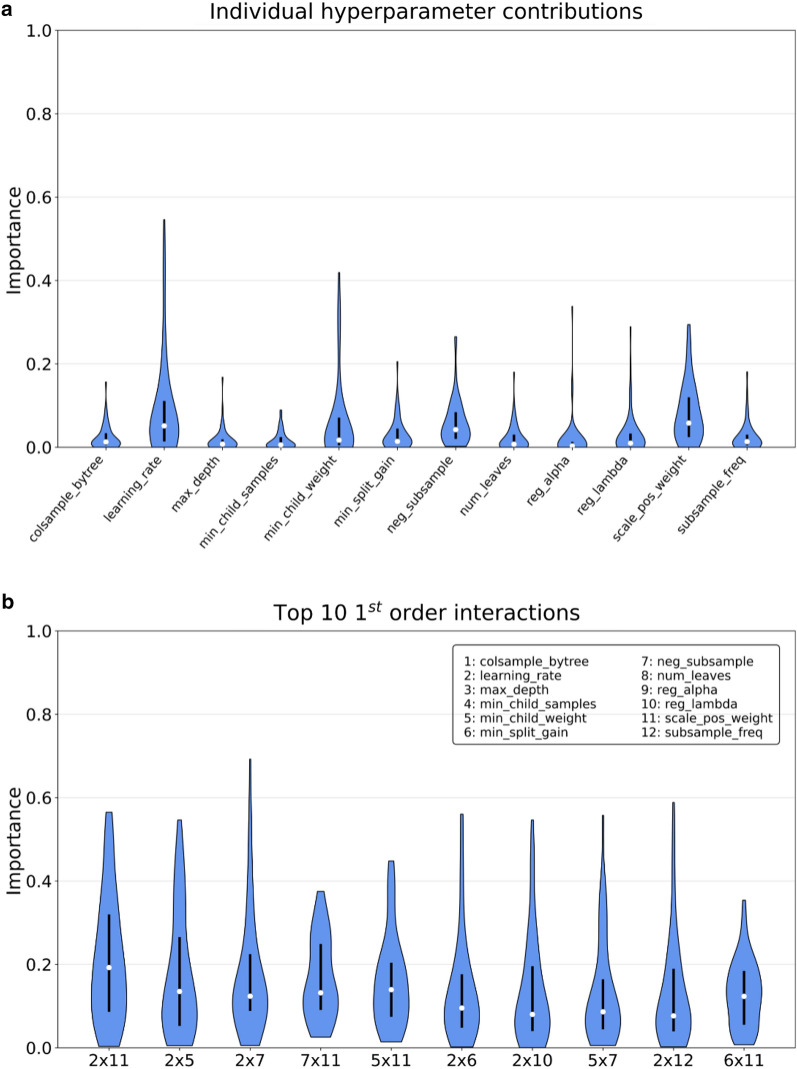
Violin plot distribution of the importance scores across all endpoints for the Tox21, MUV, HIV, BBBP, BACE, ClinTox, Phosphatase, NTPase and Oxidoreductase datasets. **a**The distribution of individual contributions for each hyperparameter, denoted by a numerical identifier. **b** The score variation of pairwise interactions. Each interaction is defined by the combination of two numeric identifiers for conciseness

After calculating the hyperparameter importance across datasets for LightGBM, we evaluated their distribution on different endpoints (Fig. 4). The analysis was limited to one GBM implementation due to the high number of optimization iterations required per endpoint. We focused our analysis on the following hyperparameters:

- “*colsample_bytree*”: fraction of features to sample at the beginning of the construction of a given tree. Tuning it helps with regularization of the ensemble.
- “*learning_rate*”: regulates how much each tree affects the overall performance of the ensemble, or in other words how many boosting rounds are required to converge. Large learning rates help with underfitting, small learning rates can help with regularization.
- “*max_depth*”: defines the maximum depth for constructing individual trees. Large values help with underfitting, small values can help with regularization.
- “*min_child_samples*”: minimum number of samples for a given leaf node. Affects tree construction and can help with regularization.
- “*min_child_weight*”: minimal sum of hessians for a given leaf node. Affects tree construction and can help with regularization.
- “*min_split_gain*”: minimal decrease in loss required to further split a node. Affects tree construction and can help with regularization.
- “*neg_subsample*”: fraction of majority class samples to use for bagging when constructing a given tree. Helps with class imbalance and regularization.
- “*num_leaves*”: Maximum number of leaves a given tree can have. Similar to max_depth but provides more fine-grained control on the shape of the tree since LightGBM uses depth-first trees.
- “*reg_alpha*”: L1 norm regularization coefficient of the leaf weights.
- “*reg_lambda*”: L2 norm regularization coefficient of the leaf weights.
- “*scale_pos_weight*”: scaling coefficient for the minority class when computing the cross-entropy loss. Large values can offset class imbalance.
- “*subsample*_freq”: affects how often to perform bagging when training the ensemble. If set to *k*, bagging is performed every *k* trees.

Generally speaking, the importance of the individual hyperparameters in the optimization process varies greatly across datasets. Furthermore, 1st order interactions between parameters play a more significant role in reaching the global optimum than tuning them in isolation, as highlighted by their larger importance score. This is consistent with the strong correlations between parameters and their non-linear effects on model behavior [39, 40, 42], which make Bayesian hyperparameter optimization necessary in the first place [51].

Looking at individual contributions (Fig. 4a), it is possible to identify highly influential hyperparameters, such as the learning rate and the minimum split gain, as well as less relevant ones, such as tree-wise feature sampling. However, all importance score distributions are remarkably skewed, highlighting that each contribution can strongly vary across different datasets. When looking at the top ten most influential pairwise interactions (Fig. 4b), most of them are related to the learning rate and the scaling coefficient for the contribution of the minority class to the global loss, highlighting the importance of tuning weighted cross-entropy when dealing with imbalanced classification. While some of these findings are consistent with the optimization guidelines from the literature, such as tuning the learning rate and the minimum split gain, others appear to contradict them. For example, while stochastic sampling of instances and features is believed to be an effective regularization technique for gradient boosting [31], in this analysis tuning it seems to be not influential in converging to the parameter configuration optimum.

To evaluate the robustness of our importance estimates, we chose to optimize LightGBM again on all datasets, tuning only the most influential parameters according to the fANOVA analysis. To do so, we selected only the parameters that appeared at least once among the top 10 most important interaction terms, yielding a grid of 7 hyperparameters instead of 12 (available in the Supporting Information). To test whether this reduced selection leads to faster convergence of the optimization process, we used 30 iterations instead of 100. As a negative control, we also evaluated the performance achieved by optimizing all hyperparameters for the same number of iterations. Finally, we expressed the ROC-AUC and PR-AUC values achieved by these benchmarks as a fraction of the performance of the optimization process with all parameters and 100 iterations. This evaluation scheme allows us to assess how well quickly tuning only the most important hyperparameters approximates the original large-scale optimization procedure.

**Fig. 5:**
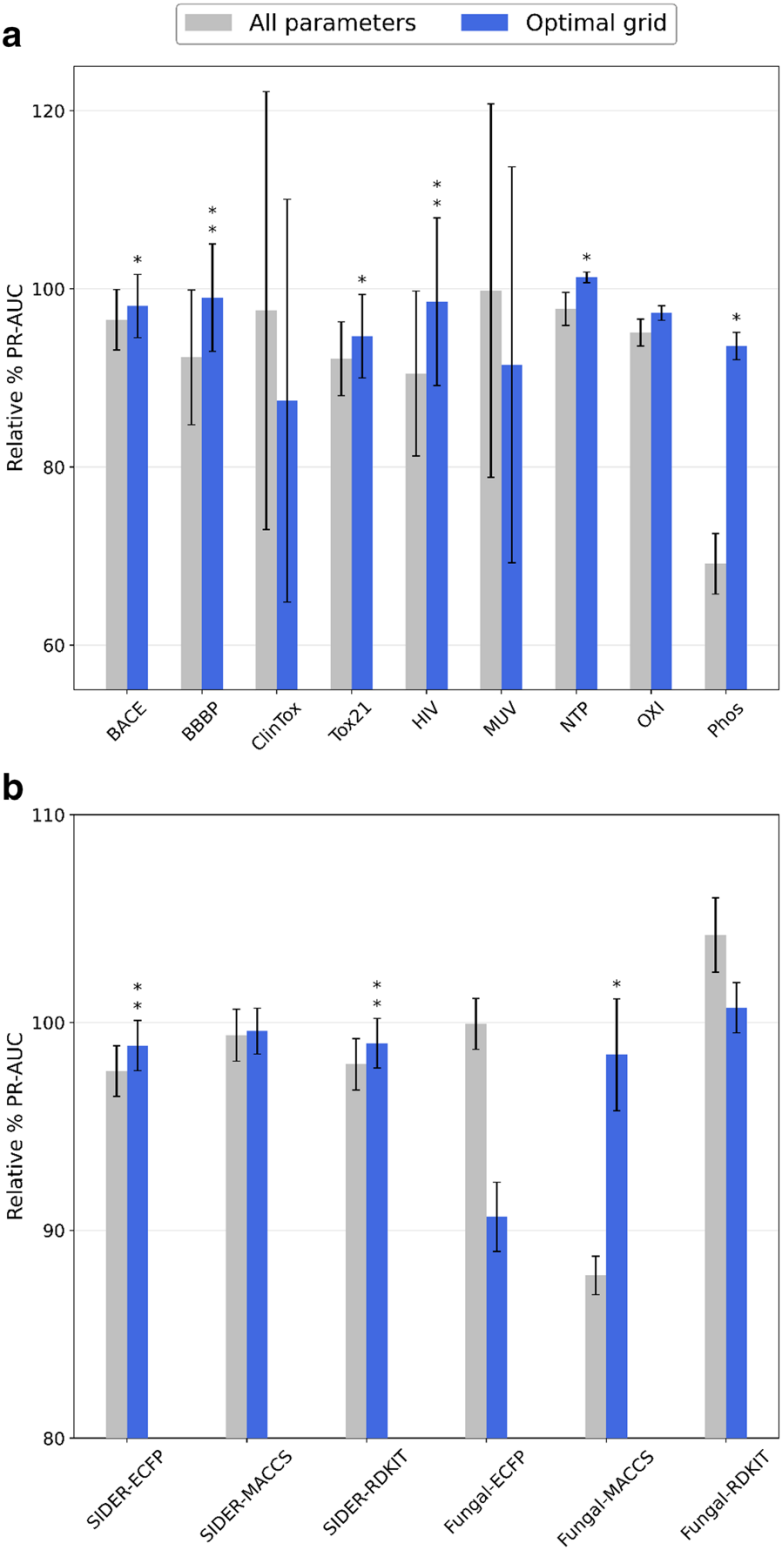
LightGBM PR-AUC comparison between carrying out hyperparameter tuning according to the optimal grid obtained from fANOVA and tuning all hyperparameters. **a** Performance on the datasets used for the fANOVA analysis. **b** Performance on the holdout datasets and with different molecular representations. Each approach was optimized for 30 iterations. The performance is reported in relation to the results obtained by tuning all parameters for 100 iterations. Error bars represent the standard deviation (*N* = *50* for MoleculeNet datasets, *N* = *5* for MolData datasets), while the asterisks denote whether the difference is significant (*: α < 0.05, **: α < 0.01, with Bonferroni correction)

As shown in Fig. 5, given the same number of iterations, using only the best parameters for the optimization process leads to consistent performance gains compared to tuning all hyperparameters. This indicates that the scores from fANOVA accurately reflect the importance of tuning a given hyperparameter for reaching the optimum. Interestingly, in some cases the optimal hyperparameter grid is able to outperform the results obtained tuning all hyperparameters for 100 iterations, such as for the NTP dataset in terms of PR-AUC and ROC-AUC (Fig. 5 and Additional file 1: Figure S2).

However, when evaluating the effectiveness of adjusting only the most important parameters on holdout datasets, the performance improvements are inconsistent. This indicates that the hyperparameter importance scores obtained by analysis of a set of endpoints do not generalize on external endpoints (Additional file 1: Figure S1). Therefore, deciding which parameters to tune must be determined on a case-by-case basis. A similar pattern is also observed when evaluating the influence of changing molecular representation for constructing the QSAR model, indicating that the parameter importance scores are highly feature-specific (Fig. 5 and Additional file 1: Figure S2).

In conclusion, optimization analysis tools such as fANOVA can be useful to further improve gradient boosting in cases where QSAR models need to be retrained periodically as new data is collected, for example for ADME prediction toolkits [3, 57]. However, the importance estimates provided by fANOVA do not generalize to unseen endpoints or different molecular representations, and limiting the optimization process to a handful of parameters can affect the performance of the classifier by up to 20%. Therefore, our recommendation is to tune all possible parameters when training gradient boosting models for QSAR, if the computational time to do so is not prohibitive. If optimizing all parameters is too costly, adjusting the learning rate, the weight of the minority class and the minimum gain to split will likely lead to the best results on a limited computational budget.

## Conclusions

This work investigated the differences between popular gradient boosting implementations in the context of cheminformatics, to guide future QSAR modelling projects. Specifically, our analysis focused on predictive performance and training time, as well as on feature ranking consistency among methods. Furthermore, we investigated which hyperparameters are the most important to tune for gradient boosting machines to reach better performance faster. To achieve these goals, we evaluated 11 different datasets, encompassing approximately 1.4 million unique compounds with a diverse selection of dataset sizes and imbalance ratios.

XGBoost generally outperformed all alternatives in terms of predictive performance by approximately 5%, at the cost of longer training times for larger datasets (*e.g.* above 100,000 compounds). LightGBM and CatBoost achieve similar performance, but the former requires significantly less time to be trained compared to the other implementations. The improvement is especially significant for datasets with more than 100,000 compounds, where LightGBM could be trained approximately 100 times faster than XGBoost and 50 times faster than CatBoost. In terms of feature importance, each implementation tends to rank molecular features differently. This not only indicates that each approach might learn slightly different structure–activity relationships, but also that caution must be exercised when using these tools to assess which fragments or properties are relevant for the biological response modelled. In this context, expert knowledge is key to critically evaluate whether these explanations could be due to chance correlation. Finally, our hyperparameter importance analysis highlights that there is significant variability in how much a given parameter affects convergence to the optimum between datasets. As such, our indication is to tune as many parameters as possible when optimizing gradient boosting models. If the computational budget is limited, our recommendation is to focus on the learning rate, the minimum split gain and the weight of the minority class if the dataset is imbalanced.

In conclusion, our study provides a set of practical guidelines for the use of gradient boosting for molecular property prediction. Given the rising popularity of this algorithm for virtual screening and QSAR, we believe our study will provide useful advice in its optimization, its use cases and limitations, thus benefitting the cheminformatics community as a whole.

### Supplementary Information

**Additional file 1: Figure S1.** Performance comparison between classification models according to ROC-AUC. **Figure S2.** LightGBM ROC-AUC comparison between carrying out hyperparameter tuning according to the optimal grid obtained from fANOVA and tuning all hyperparameters. **a)** Performance on the datasets used for the fANOVA analysis. **b)** Performance on the holdout datasets and with different molecular representations. Each approach was optimized for 30 iterations. The performance is reported in relation to the results obtained by tuning all parameters for 100 iterations. Error bars represent the standard deviation (*N=50* for MoleculeNet datasets, *N=5* for MolData datasets), while the asterisks denote whether the difference is significant (*: α<0.05, **: α<0.01, with Bonferroni correction).** Figure S3.** Top 20 most important molecular fragments according to each GBM implementation for the BACE dataset. **Table S1.** Mean number of unique substructures per compound across datasets and bit sizes.** Table S2.** List of calculated 2D molecular descriptors from the RDKIT package.

## Data Availability

The datasets and code supporting the conclusions of this article are available in the “GBM_Benchmarking” GitHub repository [https://github.com/dahvida/GBM_Benchmarking].

## Abbreviations

QSAR: Quantitative structure-activity relationship
ADME: Absorption distribution metabolism excretion
SVM: Support vector machine
GBM: Gradient boosting machine
HTS: High throughput screening
EFB: Exclusive feature bundling
GOSS: Gradient-based one sided sampling
TS: Target Statistics
ROC-AUC: Receiver operator characteristic area under curve
PR-AUC: Precision recall area under curve
RMSE: Root mean squared error
ECFP: Extended connectivity fingerprint
fANOVA: Functional analysis of variance

## References

[1] Keshavarzi Arshadi A, Salem M, Firouzbakht A, Yuan JS (2022). MolData, a molecular benchmark for disease and target based machine learning. J Cheminf.

[2] Yang K, Swanson K, Jin W, Coley C, Eiden P, Gao H, Guzman-Perez A, Hopper T, Kelley B, Mathea M, Palmer A, Settels V, Jaakkola T, Jensen K, Barzilay R (2019). Analyzing learned molecular representations for property prediction. J Chem Inf Model.

[3] Aleksić S, Seeliger D, Brown JB (2021). ADMET Predictability at Boehringer Ingelheim: state-of-the-art, and do bigger datasets or algorithms make a difference?. Mol Inform.

[4] Mayr A, Klambauer G, Unterthiner T, Steijaert M, Wegner JK, Ceulemans H, Clevert D-A, Hochreiter S (2018). Large-scale comparison of machine learning methods for drug target prediction on ChEMBL. Chem Sci.

[5] Chen H, Kogej T, Engkvist O (2018). Cheminformatics in drug discovery, an industrial perspective. Mol Inform.

[6] Withnall M, Lindelöf E, Engkvist O, Chen H (2020). Building attention and edge message passing neural networks for bioactivity and physical-chemical property prediction. J Cheminf.

[7] Santana MVS, De S-J (2021). Novo design and bioactivity prediction of sars-cov-2 main protease inhibitors using recurrent neural network-based transfer learning. BMC Chem.

[8] Gawriljuk VO, Zin PPK, Puhl AC, Zorn KM, Foil DH, Lane TR, Hurst B, Tavella TA, Costa FTM, Lakshmanane P, Bernatchez J, Godoy AS, Oliva G, Siqueira-Neto JL, Madrid PB, Ekins S (2021). Machine learning models identify inhibitors of SARS-CoV-2. J Chem Inf Model.

[9] Stokes JM, Yang K, Swanson K, Jin W, Cubillos-Ruiz A, Donghia NM, MacNair CR, French S, Carfrae LA, Bloom-Ackermann Z, Tran VM, Chiappino-Pepe A, Badran AH, Andrews IW, Chory EJ, Church GM, Brown ED, Jaakkola TS, Barzilay R, Collins JJ (2020). A deep learning approach to antibiotic discovery. Cell.

[10] Jain S, Siramshetty VB, Alves VM, Muratov EN, Kleinstreuer N, Tropsha A, Nicklaus MC, Simeonov A, Zakharov AV (2021). Large-scale modeling of multispecies acute toxicity end points using consensus of multitask deep learning methods. J Chem Inf Model.

[11] Walter M, Allen LN, de la Vega de León A, Webb SJ, Gillet VJ (2022). Analysis of the benefits of imputation models over traditional QSAR models for toxicity prediction. J Cheminf.

[12] Zhang J, Mucs D, Norinder U, Svensson F (2019). LightGBM: an effective and scalable algorithm for prediction of chemical toxicity-application to the tox21 and mutagenicity data sets. J Chem Inf Model.

[13] Grisoni F, Consonni V, Ballabio D (2019). Machine learning consensus to predict the binding to the androgen receptor within the CoMPARA project. J Chem Inf Model.

[14] Xiong G, Wu Z, Yi J, Fu L, Yang Z, Hsieh C, Yin M, Zeng X, Wu C, Lu A, Chen X, Hou T, Cao D (2021). ADMETlab 20: an integrated online platform for accurate and comprehensive predictions of ADMET properties. Nucleic Acids Res.

[15] Chuang KV, Gunsalus LM, Keiser MJ (2020). Learning molecular representations for medicinal chemistry: miniperspective. J Med Chem.

[16] Jiang D, Wu Z, Hsieh C-Y, Chen G, Liao B, Wang Z, Shen C, Cao D, Wu J, Hou T (2021). Could Graph neural networks learn better molecular representation for drug discovery? a comparison study of descriptor-based and graph-based models. J Cheminf.

[17] Winter R, Montanari F, Noé F, Clevert D-A (2019). Learning continuous and data-driven molecular descriptors by translating equivalent chemical representations. Chem Sci.

[18] Biau G, Scornet E (2016). A random forest guided tour. TEST.

[19] Lundberg SM, Erion G, Chen H, DeGrave A, Prutkin JM, Nair B, Katz R, Himmelfarb J, Bansal N, Lee S-I (2020). From local explanations to global understanding with explainable AI for trees. Nat Mach Intell.

[20] Cortes C, Vapnik V (1995). Support-vector networks. Mach Learn.

[21] Cervantes J, Garcia-Lamont F, Rodríguez-Mazahua L, Lopez A (2020). A comprehensive survey on support vector machine classification: applications. Chall Trends Neurocomp.

[22] Shwartz-Ziv R, Armon A (2022). Tabular data: deep learning is not all you need. Inf Fusion.

[23] Bentéjac C, Csörgő A, Martínez-Muñoz G (2021). A comparative analysis of gradient boosting algorithms. Artif Intell Rev.

[24] Zheng S, Aldahdooh J, Shadbahr T, Wang Y, Aldahdooh D, Bao J, Wang W, Tang J (2021). Drugcomb update: a more comprehensive drug sensitivity data repository and analysis portal. Nucleic Acids Res.

[25] Zhu Y, Brettin T, Evrard YA, Partin A, Xia F, Shukla M, Yoo H, Doroshow JH, Stevens RL (2020). Ensemble transfer learning for the prediction of anti-cancer drug response. Sci Rep.

[26] Zhang Y, Jiang Z, Chen C, Wei Q, Gu H, Yu B (2022). Deepstack-DTIs: predicting drug-target interactions using LightGBM feature selection and deep-stacked ensemble classifier. Interdiscip Sci Comput Life Sci.

[27] Wu Z, Ramsundar B, Feinberg EN, Gomes J, Geniesse C, Pappu AS, Leswing K, Pande V (2018). MoleculeNet: a benchmark for molecular machine learning. Chem Sci.

[28] Siramshetty VB, Nguyen D-T, Martinez NJ, Southall NT, Simeonov A, Zakharov AV (2020). Critical analysis. J Chem Inf Model.

[29] Boldini D, Friedrich L, Kuhn D, Sieber SA (2022). Tuning gradient boosting for imbalanced bioassay modelling with custom loss functions. J Cheminf.

[30] van Tilborg D, Alenicheva A, Grisoni F (2022). Exposing the limitations of molecular machine learning with activity cliffs. J Chem Inf Model.

[31] Chen, T.; Guestrin, C. XGBoost: A Scalable Tree Boosting System. In Proceedings of the 22nd ACM SIGKDD International Conference on Knowledge Discovery and Data Mining. ACM San Francisco California USA. 2016. 10.1145/2939672.2939785

[32] Ke G, Meng Q, Finley T, Wang T, Chen W, Ma W, Ye Q, Liu T-Y (2017). LightGBM: a highly efficient gradient boosting decision tree in advances in neural information processing systems. Curran Assoc..

[33] Prokhorenkova L, Gusev G, Vorobev A, Dorogush AV, Gulin A (2018). CatBoost: unbiased boosting with categorical features. Adv Neural Inf Process Sys.

[34] Esposito C, Landrum GA, Schneider N, Stiefl N, Riniker S (2021). GHOST: adjusting the decision threshold to handle imbalanced data in machine learning. J Chem Inf Model.

[35] Dahlin JL, Nissink JWM, Strasser JM, Francis S, Higgins L, Zhou H, Zhang Z, Walters MA (2015). PAINS in the assay: chemical mechanisms of assay interference and promiscuous enzymatic inhibition observed during a sulfhydryl-scavenging HTS. J Med Chem.

[36] Breiman L (2017). Classification and regression trees.

[37] Friedman JH (2001). Greedy function approximation: a gradient boosting machine. Ann Stat.

[38] Pedregosa F (2012). Scikit-learn: machine learning in python. Mach Learn.

[39] XGBoost Documentation—xgboost 1.6.2 documentation. https://xgboost.readthedocs.io/en/stable/. Accessed 31 Aug 2022

[40] Welcome to LightGBM’s documentation!—LightGBM 3.3.2 documentation. https://lightgbm.readthedocs.io/en/v3.3.2/. Accessed 31 Aug 2022

[41] Todeschini R, Consonni V (2000). Handbook of molecular descriptors. Methods Princ Med Chem.

[42] CatBoost - state-of-the-art open-source gradient boosting library with categorical features support. https://catboost.ai. Accessed 31 Aug 2022

[43] Ustimenko A, Beliakov A, Prokhorenkova L (2022). Gradient boosting performs gaussian process inference. ArXiv.

[44] Ustimenko, A.; Prokhorenkova, L. SGLB: Stochastic Gradient Langevin Boosting. http://arxiv.org/abs/2001.07248. Accessed 20 May 2022.

[45] Sharchilev, B.; Ustinovsky, Y.; Serdyukov, P.; de Rijke, M. Finding Influential Training Samples for Gradient Boosted Decision Trees. arXiv March 12, 2018. http://arxiv.org/abs/1802.06640 Accessed 29 Jul 2022

[46] Cortés-Ciriano I, Bender A (2019). Deep confidence: a computationally efficient framework for calculating reliable prediction errors for deep neural networks. J Chem Inf Model.

[47] Fu G, Yi L, Pan J (2019). Tuning model parameters in class-imbalanced learning with precision-recall curve. Biom J.

[48] Feng Y, Zhou M, Tong X Imbalanced classification: a paradigm-based review. http://arxiv.org/abs/2002.04592. Accessed 10 Oct 2022

[49] Dunn OJ (1961). Multiple comparisons among means. J Am Stat Assoc.

[50] RDKit. https://www.rdkit.org/. Accessed 09 May 2021

[51] Bergstra J, Komer B, Eliasmith C, Yamins D, Cox DD (2015). Hyperopt: a python library for model selection and hyperparameter optimization. Comput Sci Discov.

[52] Jiménez-Luna J, Grisoni F, Schneider G (2020). Drug discovery with explainable artificial intelligence. Nat Mach Intell.

[53] Shapley L, Kuhn HW, Tucker A (1953). A value for n-person games. Contributions to the theory of games (AM-28).

[54] Sheridan RP (2019). Interpretation of QSAR models by coloring atoms according to changes in predicted activity: how robust is it?. J Chem Inf Model.

[55] Hutter F, Hoos H, Leyton-Brown K (2014) An Efficient Approach for Assessing Hyperparameter Importance. In Proceedings of the 31st International Conference on International Conference on Machine Learning. ICML’14; JMLR.org: Beijing, China. 32:I-754–I-762. https://dl.acm.org/doi/10.5555/3044805.3044891

[56] Durant JL, Leland BA, Henry DR, Nourse JG (2002). Reoptimization of MDL keys for use in drug discovery. J Chem Inf Model.

[57] Göller AH, Kuhnke L, Montanari F, Bonin A, Schneckener S, ter Laak A, Wichard J, Lobell M, Hillisch A (2020). Bayer’s in silico ADMET platform: a journey of machine learning over the past two decades. Drug Discov Today.

