# Supplementary material for: Practical guidelines for the use of gradient boosting for molecular property prediction
Source: J Cheminform. 2023 Aug 28;15:73. doi: 10.1186/s13321-023-00743-7 (PMC10464382; doi:10.1186/s13321-023-00743-7)
Supplement: Supplementary file 1 — Additional file 1: Figure S1. Performance comparison between classification models according to ROC-AUC. Figure S2. LightGBM ROC-AUC comparison between carrying out hyperparameter tuning according to the optimal grid obtained from fANOVA and tuning all hyperparameters. a) Performance on the datasets used for the fANOVA analysis. b) Performance on the holdout datasets and with different molecular representations. Each approach was optimized for 30 iterations. The performance is reported in relation to the results obtained by tuning all parameters for 100 iterations. Error bars represent the standard deviation (N=50 for MoleculeNet datasets, N=5 for MolData datasets), while the asterisks denote whether the difference is significant (*: α<0.05, **: α<0.01, with Bonferroni correction). Figure S3. Top 20 most important molecular fragments according to each GBM implementation for the BACE dataset. Table S1. Mean number of unique substructures per compound across datasets and bit sizes. Table S2. List of calculated 2D molecular descriptors from the RDKIT package. [file 13321_2023_743_MOESM1_ESM.docx]

**Additional Information**

Practical guidelines for the use of gradient boosting for molecular property prediction

Davide Boldini,^†^ Francesca Grisoni,^‡,∥^ Daniel Kuhn,^§^ Lukas Friedrich^§^ and Stephan A. Sieber*^,†^

† Department of Bioscience, Center for Functional Protein Assemblies (CPA), Technical University of Munich, Garching bei München, Germany

‡ Department of Biomedical Engineering, Institute for Complex Molecular Sciences, Eindhoven University of Technology, Eindhoven, Netherlands

∥ Centre for Living Technologies, Alliance TU/e, WUR, UU, UMC Utrecht, Netherlands

§ Merck Healthcare KGaA, Darmstadt, Germany

1. **Hyperparameter grids**

**XGBoost:**

- max_leaves: discrete loguniform [1, 4]
- learning_rate: discrete loguniform [-3, 0]
- max_depth: discrete uniform [3, 12]
- min_child_sample: discrete loguniform [0, 5]
- min_child_weight: loguniform [-5, 5]
- reg_alpha: loguniform [-5, 2]
- reg_lambda: loguniform [-5, 2]
- max_delta_step: loguniform [-5, 2]
- colsample_bytree: uniform [0.1, 1]
- colsample_bylevel: uniform [0.1, 1]
- subsample: uniform [0.2, 1]
- scale_pos_weight: loguniform [0, 6]

**LightGBM:**

- colsample_bytree: uniform [0.1, 1]
- learning_rate: discrete loguniform [-3, 0]
- max_depth: discrete uniform [3, 12]
- min_child_samples: discrete loguniform [0, 5]
- min_child_weight: loguniform [-5, 5]
- min_split_gain: uniform [0, 15]
- neg_subsample: uniform [0.1, 1]
- num_leaves: discrete loguniform [1, 5]
- reg_alpha: loguniform [-5, 2]
- reg_lambda: loguniform [-5, 2]
- scale_pos_weight: loguniform [0, 6]
- subsample_freq: discrete uniform [0, 30]

**CatBoost:**

- learning_rate: discrete loguniform [-3, 0]
- depth: discrete uniform [3, 12]
- leaf_estimation_iterations: discrete uniform [1, 20]
- l2_leaf_reg: loguniform [-5, 2]
- random_strength: discrete uniform [1, 20]
- subsample: uniform [0.1, 1]
- sampling_frequency: choice [PerTree, PerTreeLevel]
- colsample_bylevel: uniform [0.1, 1]
- scale_pos_weight: loguniform [0, 6]
- langevin: choice [True, False]

For all GBM implementations, the number of iterations was optimized via early stopping monitoring the loss on the validation set instead of treating it as an additional hyperparametrer.

1. **LightGBM hyperparameter definition**

- **colsample_bytree:** fraction of features to sample at the beginning of the construction of a given tree. Tuning it helps with regularization of the ensemble.
- **learning_rate**: regulates how much each tree affects the overall performance of the ensemble, or in other words how many boosting rounds are required to converge. Large learning rates help with underfitting, small learning rates can help with regularization.
- **max_depth:** defines the maximum depth for constructing individual trees. Large values help with underfitting, small values can help with regularization.
- **min_child_samples:** minimum number of samples for a given leaf node. Affects tree construction and can help with regularization.
- **min_child_weight:** minimal sum of hessians for a given leaf node. Affects tree construction and can help with regularization.
- **min_split_gain:** minimal decrease in loss required to further split a node. Affects tree construction and can help with regularization.
- **neg_subsample:** fraction of majority class samples to use for bagging when constructing a given tree. Helps with class imbalance and regularization.
- **num_leaves:** Maximum number of leaves a given tree can have. Similar to max_depth but provides more fine-grained control on the shape of the tree since LightGBM uses depth-first trees.
- **reg_alpha:** L1 norm regularization coefficient of the leaf weights.
- **reg_lambda:** L2 norm regularization coefficient of the leaf weights.
- **scale_pos_weight:** scaling coefficient for the minority class when computing the cross-entropy loss. Large values can offset class imbalance.
- **subsample_freq:** affects how often to perform bagging when training the ensemble. If set to *k*, bagging is performed every *k* trees.

1. **LightGBM optimal hyperparameter grid**

- learning_rate: discrete loguniform [-3, 0]
- min_child_weight: loguniform [-5, 5]
- min_split_gain: uniform [0, 15]
- neg_subsample: uniform [0.1, 1]
- reg_lambda: loguniform [-5, 2]
- scale_pos_weight: loguniform [0, 6]
- subsample_freq: discrete uniform [0, 30]

1. **Fingerprint density analysis**

**Table S1** – Mean number of unique substructures per compound across datasets and bit sizes.

| **Dataset** | **1024 bits** | **2048 bits** | **4096 bits** |
| --- | --- | --- | --- |
| bace | 60.069397 | 60.6549911 | 61.3615341 |
| bbbp | 42.1981354 | 42.7047577 | 43.2731743 |
| clintox | 43.2341003 | 43.7293625 | 44.2131271 |
| hiv | 38.9629936 | 39.3100014 | 39.698143 |
| muv | 44.1438484 | 44.5432358 | 44.8428497 |
| ntp | 47.7583199 | 48.243885 | 48.6402512 |
| oxi | 47.7583199 | 48.243885 | 48.6402512 |
| phos | 47.7583199 | 48.243885 | 48.6402512 |
| tox21 | 29.582428 | 29.8293953 | 30.1690712 |
| ache | 48.0531807 | 48.6305809 | 48.9977837 |
| cox2 | 42.7222404 | 43.0679512 | 43.5656738 |
| erbb1 | 54.7732124 | 55.2356186 | 55.6382484 |
| herg | 55.9122353 | 56.4770508 | 56.953907 |
| jak2 | 56.2000008 | 56.6851234 | 57.0817337 |
| fungal | 47.7583199 | 48.243885 | 48.6402512 |
| sider | 45.7918701 | 46.5136642 | 47.2221451 |

1. **RDKIT Molecular descriptors**

**Table S2** – List of calculated 2D molecular descriptors from the RDKIT package.

| **ID** | **Name** | **ID** | **Name** |
| --- | --- | --- | --- |
| 1 | MaxEStateIndex | 105 | HeavyAtomCount |
| 2 | MinEStateIndex | 106 | NHOHCount |
| 3 | MaxAbsEStateIndex | 107 | NOCount |
| 4 | MinAbsEStateIndex | 108 | NumAliphaticCarbocycles |
| 5 | qed | 109 | NumAliphaticHeterocycles |
| 6 | MolWt | 110 | NumAliphaticRings |
| 7 | HeavyAtomMolWt | 111 | NumAromaticCarbocycles |
| 8 | ExactMolWt | 112 | NumAromaticHeterocycles |
| 9 | NumValenceElectrons | 113 | NumAromaticRings |
| 10 | NumRadicalElectrons | 114 | NumHAcceptors |
| 11 | MaxPartialCharge | 115 | NumHDonors |
| 12 | MinPartialCharge | 116 | NumHeteroatoms |
| 13 | MaxAbsPartialCharge | 117 | NumRotatableBonds |
| 14 | MinAbsPartialCharge | 118 | NumSaturatedCarbocycles |
| 15 | FpDensityMorgan1 | 119 | NumSaturatedHeterocycles |
| 16 | FpDensityMorgan2 | 120 | NumSaturatedRings |
| 17 | FpDensityMorgan3 | 121 | RingCount |
| 18 | BCUT2D_MWHI | 122 | MolLogP |
| 19 | BCUT2D_MWLOW | 123 | MolMR |
| 20 | BCUT2D_CHGHI | 124 | fr_Al_COO |
| 21 | BCUT2D_CHGLO | 125 | fr_Al_OH |
| 22 | BCUT2D_LOGPHI | 126 | fr_Al_OH_noTert |
| 23 | BCUT2D_LOGPLOW | 127 | fr_ArN |
| 24 | BCUT2D_MRHI | 128 | fr_Ar_COO |
| 25 | BCUT2D_MRLOW | 129 | fr_Ar_N |
| 26 | BalabanJ | 130 | fr_Ar_NH |
| 27 | BertzCT | 131 | fr_Ar_OH |
| 28 | Chi0 | 132 | fr_COO |
| 29 | Chi0n | 133 | fr_COO2 |
| 30 | Chi0v | 134 | fr_C_O |
| 31 | Chi1 | 135 | fr_C_O_noCOO |
| 32 | Chi1n | 136 | fr_C_S |
| 33 | Chi1v | 137 | fr_HOCCN |
| 34 | Chi2n | 138 | fr_Imine |
| 35 | Chi2v | 139 | fr_NH0 |
| 36 | Chi3n | 140 | fr_NH1 |
| 37 | Chi3v | 141 | fr_NH2 |
| 38 | Chi4n | 142 | fr_N_O |
| 39 | Chi4v | 143 | fr_Ndealkylation1 |
| 40 | HallKierAlpha | 144 | fr_Ndealkylation2 |
| 41 | Ipc | 145 | fr_Nhpyrrole |
| 42 | Kappa1 | 146 | fr_SH |
| 43 | Kappa2 | 147 | fr_aldehyde |
| 44 | Kappa3 | 148 | fr_alkyl_carbamate |
| 45 | LabuteASA | 149 | fr_alkyl_halide |
| 46 | PEOE_VSA1 | 150 | fr_allylic_oxid |
| 47 | PEOE_VSA10 | 151 | fr_amide |
| 48 | PEOE_VSA11 | 152 | fr_amidine |
| 49 | PEOE_VSA12 | 153 | fr_aniline |
| 50 | PEOE_VSA13 | 154 | fr_aryl_methyl |
| 51 | PEOE_VSA14 | 155 | fr_azide |
| 52 | PEOE_VSA2 | 156 | fr_azo |
| 53 | PEOE_VSA3 | 157 | fr_barbitur |
| 54 | PEOE_VSA4 | 158 | fr_benzene |
| 55 | PEOE_VSA5 | 159 | fr_benzodiazepine |
| 56 | PEOE_VSA6 | 160 | fr_bicyclic |
| 57 | PEOE_VSA7 | 161 | fr_diazo |
| 58 | PEOE_VSA8 | 162 | fr_dihydropyridine |
| 59 | PEOE_VSA9 | 163 | fr_epoxide |
| 60 | SMR_VSA1 | 164 | fr_ester |
| 61 | SMR_VSA10 | 165 | fr_ether |
| 62 | SMR_VSA2 | 166 | fr_furan |
| 63 | SMR_VSA3 | 167 | fr_guanido |
| 64 | SMR_VSA4 | 168 | fr_halogen |
| 65 | SMR_VSA5 | 169 | fr_hdrzine |
| 66 | SMR_VSA6 | 170 | fr_hdrzone |
| 67 | SMR_VSA7 | 171 | fr_imidazole |
| 68 | SMR_VSA8 | 172 | fr_imide |
| 69 | SMR_VSA9 | 173 | fr_isocyan |
| 70 | SlogP_VSA1 | 174 | fr_isothiocyan |
| 71 | SlogP_VSA10 | 175 | fr_ketone |
| 72 | SlogP_VSA11 | 176 | fr_ketone_Topliss |
| 73 | SlogP_VSA12 | 177 | fr_lactam |
| 74 | SlogP_VSA2 | 178 | fr_lactone |
| 75 | SlogP_VSA3 | 179 | fr_methoxy |
| 76 | SlogP_VSA4 | 180 | fr_morpholine |
| 77 | SlogP_VSA5 | 181 | fr_nitrile |
| 78 | SlogP_VSA6 | 182 | fr_nitro |
| 79 | SlogP_VSA7 | 183 | fr_nitro_arom |
| 80 | SlogP_VSA8 | 184 | fr_nitro_arom_nonortho |
| 81 | SlogP_VSA9 | 185 | fr_nitroso |
| 82 | TPSA | 186 | fr_oxazole |
| 83 | EState_VSA1 | 187 | fr_oxime |
| 84 | EState_VSA10 | 188 | fr_para_hydroxylation |
| 85 | EState_VSA11 | 189 | fr_phenol |
| 86 | EState_VSA2 | 190 | fr_phenol_noOrthoHbond |
| 87 | EState_VSA3 | 191 | fr_phos_acid |
| 88 | EState_VSA4 | 192 | fr_phos_ester |
| 89 | EState_VSA5 | 193 | fr_piperdine |
| 90 | EState_VSA6 | 194 | fr_piperzine |
| 91 | EState_VSA7 | 195 | fr_priamide |
| 92 | EState_VSA8 | 196 | fr_prisulfonamd |
| 93 | EState_VSA9 | 197 | fr_pyridine |
| 94 | VSA_EState1 | 198 | fr_quatN |
| 95 | VSA_EState10 | 199 | fr_sulfide |
| 96 | VSA_EState2 | 200 | fr_sulfonamd |
| 97 | VSA_EState3 | 201 | fr_sulfone |
| 98 | VSA_EState4 | 202 | fr_term_acetylene |
| 99 | VSA_EState5 | 203 | fr_tetrazole |
| 100 | VSA_EState6 | 204 | fr_thiazole |
| 101 | VSA_EState7 | 205 | fr_thiocyan |
| 102 | VSA_EState8 | 206 | fr_thiophene |
| 103 | VSA_EState9 | 207 | fr_unbrch_alkane |
| 104 | FractionCSP3 | 208 | fr_urea |

1. **Additional figures**


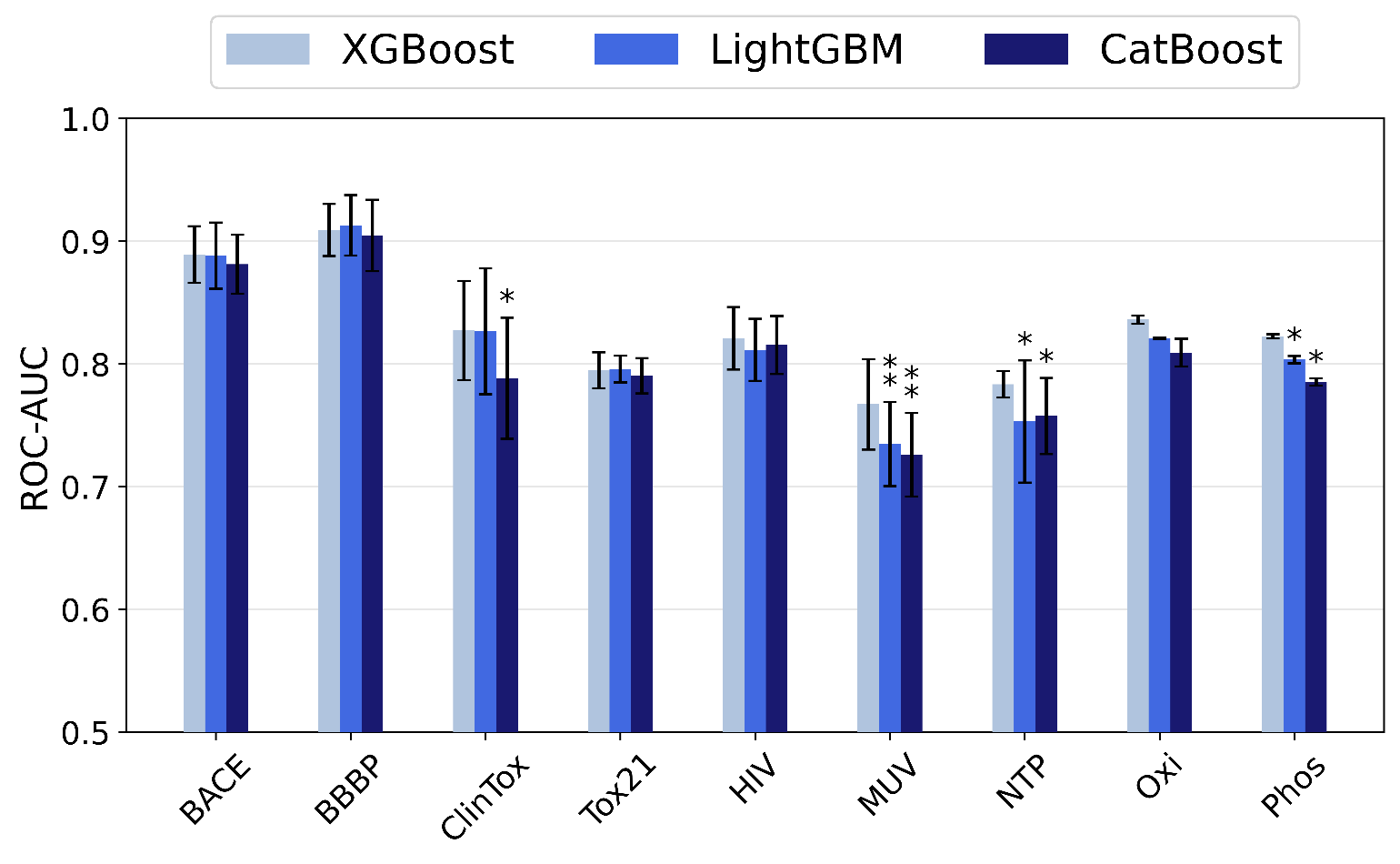


**Figure S1** – Performance comparison between classification models according to ROC-AUC.


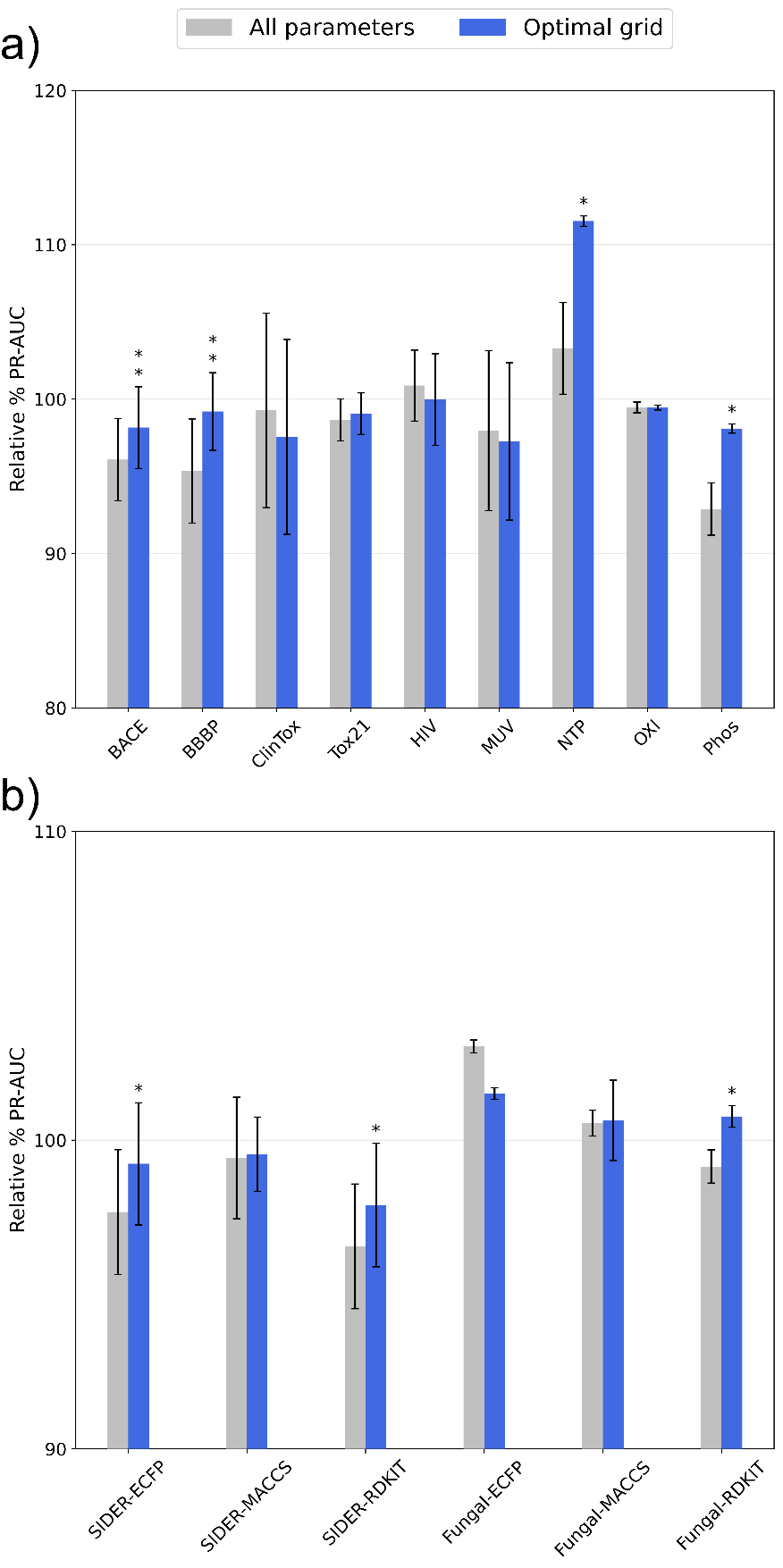


**Figure S2** - LightGBM ROC-AUC comparison between carrying out hyperparameter tuning according to the optimal grid obtained from fANOVA and tuning all hyperparameters. a) Performance on the datasets used for the fANOVA analysis. b) Performance on the holdout datasets and with different molecular representations. Each approach was optimized for 30 iterations. The performance is reported in relation to the results obtained by tuning all parameters for 100 iterations. Error bars represent the standard deviation (*N=50* for MoleculeNet datasets, *N=5* for MolData datasets), while the asterisks denote whether the difference is significant (*: α<0.05, **: α<0.01, with Bonferroni correction).


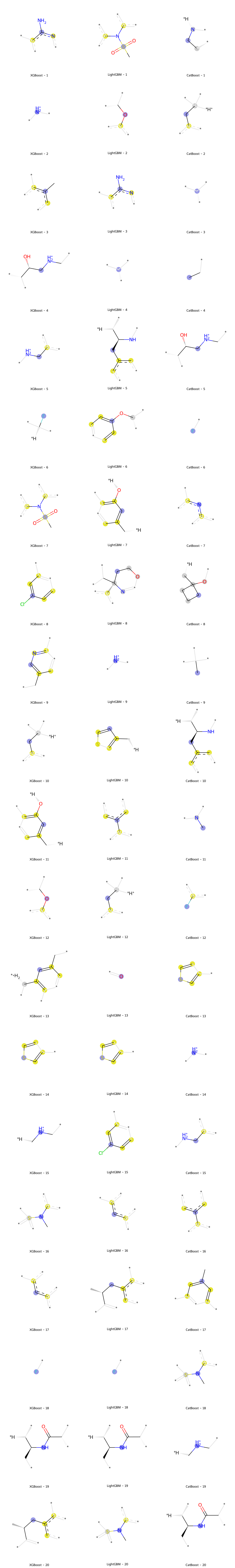


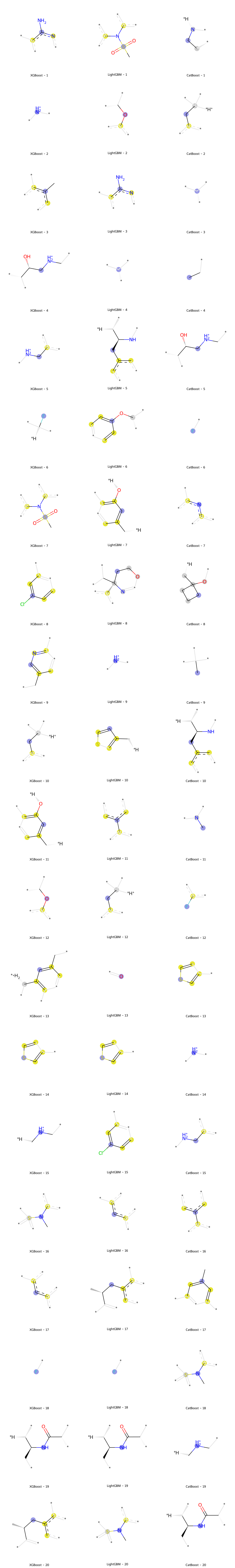


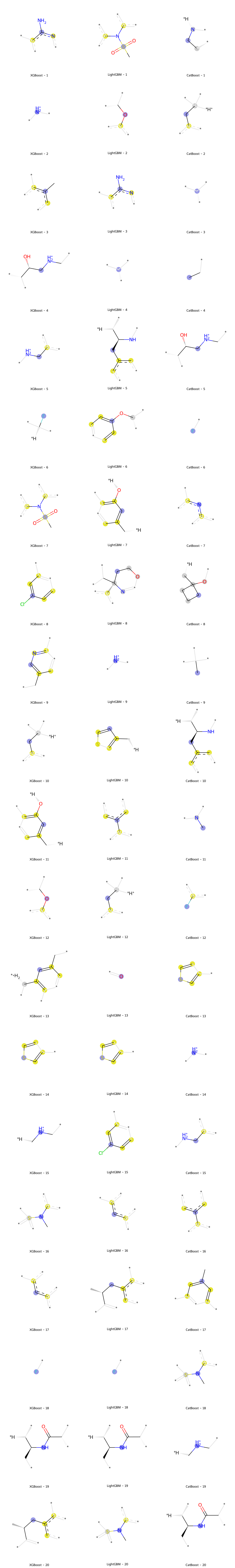


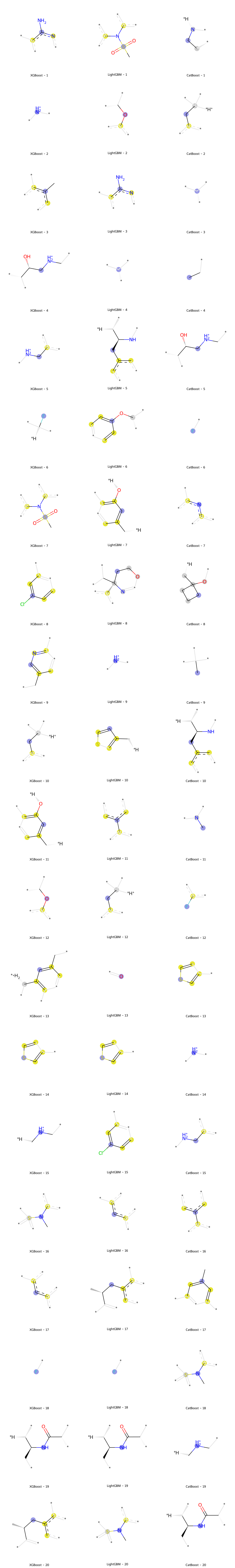


**Figure S3** – Top 20 most important molecular fragments according to each GBM implementation for the BACE dataset.
